# Impact of Incretin-Based Therapy on Skeletal Muscle Health

**DOI:** 10.3390/medicina61091691

**Published:** 2025-09-18

**Authors:** Andrijana Koceva, Andrej Janež, Mojca Jensterle

**Affiliations:** 1 Department of Endocrinology and Diabetology, University Medical Center Maribor, 2000 Maribor, Slovenia; 2 Faculty of Medicine, University of Maribor, 2000 Maribor, Slovenia; 3 Department of Endocrinology, Diabetes and Metabolic Diseases, University Medical Center Ljubljana, 1000 Ljubljana, Slovenia; 4 Faculty of Medicine, University of Ljubljana, 1000 Ljubljana, Slovenia

**Keywords:** obesity, incretin-based treatment, liraglutide, semaglutide, tirzepatide, muscle quality, muscle function

## Abstract

Skeletal muscle is the largest insulin-sensitive tissue in the human body, playing a crucial role in glucose homeostasis, body mobility and overall metabolic health. In obesity and type 2 diabetes (T2D), skeletal muscle undergoes structural, functional, and metabolic alterations, including reduced muscle mass, impaired contractile function, increased myosteatosis, mitochondrial dysfunction, and chronic low-grade inflammation. Incretin-based therapies such as glucagon-like peptide-1 receptor agonists (GLP-1 RAs) or dual GLP-1/glucose-dependent insulinotropic polypeptide (GIP) RAs are highly effective treatments for T2D and obesity, producing substantial weight loss. While clinical trials suggest proportional loss of fat and lean mass when using incretin-based therapies, emerging preclinical and translational data indicate potential muscle-specific beneficial effects such as attenuation of atrophy, improved myogenesis, enhanced mitochondrial function and reduced myosteatosis. This review comprehensively summarizes the current preclinical and clinical evidence on the impact of incretin-based therapies on skeletal muscle mass, composition, metabolism, and performance, focusing on mechanistic insights from animal models and translational findings from human studies.

## 1. Introduction

Skeletal muscle is the largest metabolically active, insulin-sensitive tissue, accounting for approximately 33 to 50% of whole-body protein turnover and 75% of postprandial glucose disposal [1,2]. Muscle mass typically increases during childhood and adolescence, peaks between 30 and 40 years of age, and then progressively declines. This natural decline is accelerated in T2D and low muscle mass is strongly linked to insulin resistance, frailty and sarcopenia [2,3,4]. Obesity further compounds these effects by impairing muscle quality and function, partly through a shift in fiber composition from oxidative Type I to more glycolytic Type II fibers, reducing oxidative capacity and metabolic flexibility [5]. In addition, obesity and T2D promote chronic low-grade inflammation, characterized by elevated inflammatory factors such as TNF-α, IL-6 and IL-1β. This pro-inflammatory environment promotes insulin resistance, accelerates muscle protein degradation, and suppresses muscle fiber differentiation [5].

Myosteatosis, characterized by intermuscular, intramuscular and intramyocellular fat infiltration, is another pathological feature linked to insulin resistance, metabolic dysfunction and impaired muscle performance. It plays an important role in the development of sarcopenia and is often observed in older adults, malignancies and metabolic diseases, such as diabetes and obesity [6].

Incretin-based therapies have become central to obesity and T2D care. GLP-1 receptor agonists such as liraglutide (3 mg daily) and semaglutide (2.4 mg weekly) are registered for chronic weight management, producing a mean weight loss of 8% and from 14.9% to 16%, respectively [7,8,9]. Tirzepatide is the first dual GLP-1/GIP receptor agonist registered for chronic weight management with a reported mean weight loss of 15% (5 mg), 19.5% (10 mg), and 20.9% (15 mg) [10].

While these agents induce substantial weight loss and improve cardiometabolic outcomes, the reduction in body weight also involves a decrease in fat-free mass, raising concerns about potential sarcopenia, particularly in vulnerable populations. Conversely, preclinical in-vitro and in-vivo studies suggest that incretin-based therapies may directly benefit skeletal muscle quality beyond their weight and glucose-lowering properties. In this narrative review, we comprehensively summarize the current preclinical and clinical evidence on the impact of incretin-based therapies on skeletal muscle mass, composition, metabolism, and performance, highlighting mechanistic insights from animal models and translational findings from human studies.

## 2. Materials and Methods

We searched the PubMed database using a combination of MeSH terms and keywords, such as glucagon-like peptide-1 receptor agonists, GLP-1 receptor agonist, GLP-1/GIP receptor agonist, liraglutide, semaglutide or tirzepatide in combination with other terms such as muscle, muscle mass, lean mass, fat-free mass, lean body mass, sarcopenia, muscle strength, muscle function, muscle performance. The search was limited to articles written in English over the past 20 years (from July 2005 to July 2025). Additional studies were identified by analysing the reference lists. We included studies reporting an outcome related to muscle health and muscle mass, including preclinical interventional studies, case studies, clinical trials, randomized controlled trials, observational studies and meta-analysis. The initial search included 303 articles, including human data from 38 articles and animal data from 24 articles.

## 3. Impact of Incretin-Based AOMs on Skeletal Muscle—Data from Animal Studies

Skeletal muscle mass is determined by the balance between protein synthesis, predominantly driven by the Akt/mTOR pathway and protein breakdown through the ubiquitin-proteasome system and autophagy-lysosome pathway [11]. Mitochondrial biogenesis, primarily regulated by the transcriptional coactivator peroxisome proliferator-activated receptor gamma coactivator 1-α (PGC-1α), links oxidative capacity to protein homeostasis, while emerging regenerative pathways (Wnt, Notch, and Hippo/YAP-TAZ) further contribute to myogenesis and structural remodeling [11]. In preclinical models, incretin-based therapies have been shown to modulate several of these pathways, providing mechanistic insights into their potential role in muscle health beyond glucose and weight regulation.

### 3.1. Glucose Metabolism and Insulin Sensitivity

Glucose metabolism and insulin sensitivity in skeletal muscle depend on insulin receptor (IR) activation, phosphatidylinositol 3-kinase (PI3K)/Akt signaling, and glucose transporters (GLUTs) translocation [12]. GLUT4, the predominant insulin-responsive glucose transporter in muscles, is primarily found in intracellular storage vesicles and translocated to the plasma membrane in response to downstream Akt activation of the PI3K/Akt pathway [13]. On the other hand, protein tyrosine phosphatase 1B (PTP1B) is an upstream, negative regulator of this pathway, dephosphorylating IR and its substrates, thereby inhibiting the PI3K/Akt, reducing GLUT4 translocation, glucose utilization and protein synthesis. In obesity and diabetes, skeletal muscle PI3K/Akt signaling is impaired, while PTP1B expression is elevated, contributing to insulin resistance and muscle atrophy [14,15,16].

KK-Ay mice, which spontaneously develop obesity and hyperglycemia, are often used to investigate these pathways. In this model, Ji et al. demonstrated that liraglutide downregulates PTP1B expression and upregulates PI3K expression, thereby increasing PI3K/Akt2 signaling, resulting in greater GLUT4 translocation and improved glucose metabolism [16]. Similar findings were reported for both liraglutide and semaglutide across various diabetic and obesity mouse models [12,17,18,19]. Additionally, liraglutide enhances glycolytic enzyme activity (hexokinase and pyruvate kinase), improving skeletal muscle glucose utilization and insulin sensitivity [12].

Intramuscular lipid accumulation, another hallmark of insulin resistance, is also attenuated by liraglutide [16]. Mechanistically, a lipotoxic environment in skeletal muscle cells drives serine phosphorylation of insulin receptor substrate-1 (IRS-1), impairing its ability to undergo tyrosine phosphorylation and thereby blocking downstream Akt and insulin signaling. In palmitate-induced lipotoxicity models, liraglutide counteracted lipotoxicity, likely by suppressing IKKα/β and JNK activity [20] and activating SENS2, a stress-inducible protein with antioxidant and autophagy-promoting properties [13], restoring IRS-1 tyrosine phosphorylation and reactivating the insulin signaling cascade.

Lastly, insulin promotes glucose uptake not only through metabolic signaling but also by increasing microvascular recruitment, which enhances the delivery of oxygen and glucose into the muscle [21]. This insulin-mediated microvascular recruitment and skeletal muscle capillary density are decreased in insulin-resistant states. In both rodents and humans, GLP-1 significantly increases microvascular recruitment and muscle capillary density, improving microvascular insulin sensitivity even in insulin-resistant states [21,22,23]. In contrast, recent human studies suggest that elevated GIP levels during oral glucose challenge negatively correlate with skeletal muscle microvascular blood flow, possibly via endothelin-1-mediated vasoconstriction. This highlights that the role of GIP in regulating skeletal muscle microvascular tone remains poorly understood, yet could be pivotal in modulating muscle perfusion and insulin sensitivity [23].

These findings indicate that by restoring PI3K/Akt signaling, enhancing GLUT4 translocation, increasing microvascular recruitment, reducing intramuscular lipid accumulation and lipotoxic signaling, GLP-1 RAs can enhance skeletal muscle glucose uptake and improve metabolic flexibility in patients with an insulin-resistant state, such as obesity and T2D.

### 3.2. Muscle Mass, Atrophy and Regeneration

Beyond metabolic modulation, GLP-1 RAs have significantly influenced skeletal muscle mass, structure, and regenerative capacity in preclinical in vivo and in vitro models. Multiple studies demonstrate that GLP-1 RA, such as liraglutide and semaglutide, can exert protective and regenerative effects on skeletal muscle.

Muscle atrophy, a significant complication associated with diabetes, is characterized by reduced muscle protein synthesis and increased protein degradation, partly mediated by activation of the ubiquitin protease system with elevated E3 ubiquitin ligases atrogin-1 (MAFbx) and MuRF1 [24].

In diabetic and high-fat diet mouse models, liraglutide and semaglutide increase muscle mass, fiber size and density, restore strength and contractile function, while also influencing muscle quality by reducing lipid infiltration and improving fiber morphology [5,19,25]. In a muscle atrophy induced by diethoxycarbonyl-1,4-dihydrocollidine (DDC) diet, semaglutide also preserved grip strength and attenuated wasting [26]. These protective effects of GLP-1 RAs have also been confirmed in multiple other in vitro (C2C12 cells) and in vivo atrophy models (freeze injury, denervation, dexamethasone-induced muscle atrophy, ovariectomy-induced atrophy) [27].

Mechanistically, GLP-1 RAs suppress ubiquitin-mediated skeletal muscle protein degradation markers (atrogin-1 and MuRF-1) and increase myogenic factors (MyoD and MyoG) [24,26]. These changes are accompanied by improved myofibrillar structure, cross-striation restoration, enhanced contractile activity and improved glucose utilization [16,27,28]. GLP-1 RAs also engage the YAP/TAZ pathway, a crucial pathway for regulating tissue size and aging [25], activate sirtuin 1 (SIRT1), a key regulator of skeletal muscle metabolism, mitochondrial function, and regeneration [19], and stimulate cAMP-dependent myoblast differentiation and myogenesis [27].

Transcriptomic profiling of cultured mouse skeletal C2C12 myoblasts under hyperglycemic conditions further supported these mechanisms by showing that liraglutide reverses several detrimental molecular signatures, including AMPK downregulation (a key regulator of energy balance), atrogen upregulation (key regulators of muscle protein degradation), increased 3-methylhistidine expression (a byproduct of actin and myosin degradation) and myogenic gene downregulation, indicating preserved myotube integrity and regeneration [29].

The ability of GLP-1 RAs to suppress muscle protein breakdown and promote regenerative pathways in animal models suggests that GLP-1 RAs may help preserve lean mass during weight loss or catabolic states.

### 3.3. Mitochondrial Function, Oxidative Capacity and Muscle-Adipose Crosstalk

Mitochondrial dysfunction is a known trigger of skeletal muscle atrophy, impairing oxidative phosphorylation, fatty acid oxidation and energy homeostasis. Several preclinical studies show that GLP-1 RAs counteract these deficits by impacting mitochondrial biogenesis, oxidative metabolism and inter-tissue communication.

In diethoxycarbonyl-1,4-dihydrocollidine (DDC) diet-fed KK-Ay mice, a model of skeletal muscle atrophy, semaglutide restored intramuscular mRNA and protein levels of PGC-1α, mitochondrial transcription factor A (mtTFA), and sirtuin 1 (SIRT1), key regulators of mitochondrial biogenesis [26]. Liraglutide produced comparable effects, increasing mitochondrial number by 441%, individual mitochondrial area by 113%, total mitochondrial area by 396% in diabetic mice, alongside improved mitochondrial morphology [16]. Similar increases in mitochondrial number and area have been observed with semaglutide in HFD-fed mice [5].

Beyond quantity, GLP-1 RAs promote oxidative metabolism and thermogenic reprogramming. In high-fat/high-sucrose (HFHS) diet-fed mice, liraglutide upregulates PRDM16 and brown fat markers (UCP-1, PPARα) in the skeletal muscle [30] and suppresses lipogenic regulators (PPARγ, C/EBPα) in muscle, liver and perigonadal adipose tissue, indicating coordinated inhibition of adipogenesis and improved insulin sensitivity [30]. It also enhances fatty acid oxidation by increasing long-chain acyl-CoA dehydrogenase expression and reducing acetyl-CoA carboxylase phosphorylation in skeletal muscle [30].

GLP-1 RAs further promote oxidative metabolism through upregulation of PGC-1α in both skeletal muscle and white adipose tissue, supporting enhanced mitochondrial biogenesis, mitochondrial capacity and fatty acid oxidation even in nutrient-excess states [31]. This enhanced oxidative phosphorylation and mitochondrial efficiency were confirmed in both high-fat diet (HFD)-fed and diabetic mice models [32,33].

GLP-1 RA treatment may also preserve skeletal muscle functional capacity. In a study comparing vertical sleeve gastrectomy (VSG) and semaglutide in obese male mice, both VSG and semaglutide comparably reduced body weight and improved glucose and lipid metabolism, but only semaglutide preserved energy expenditure normalized to lean mass and increased ambulatory nighttime activity [34], suggesting potential benefits in maintaining skeletal muscle metabolic activity and performance.

Muscle-adipose crosstalk may contribute to these effects. In cultured mouse skeletal muscle cells, liraglutide was found to increase irisin secretion, inducing UCP-1 expression, lipolysis and adipose tissue browning in cultured white fat cells [35]. Liraglutide-induced skeletal muscle PRDM16 upregulation and increased PPARα expression further support bidirectional transdifferentiation towards a more oxidative and thermogenic muscle phenotype [30,35]. These thermogenic shifts are diet dependent, most evident under HFHS feeding [30].

Finally, at a metabolomic level, in times of HFD, liraglutide remodels skeletal muscle metabolism by reducing fatty acids and partially restoring carbohydrate and amino acid metabolism toward a lean, non-obese phenotype [36], an adaptation with clinical relevance given the association between intramuscular fat and cardiovascular risk, heart failure and reduced functional capacity [37].

Enhancing mitochondrial health and shifting toward oxidative and thermogenic muscle phenotypes suggests GLP-1 RAs could help maintain energy expenditure, endurance, and metabolic efficiency during weight loss, potentially reducing the decline in functional capacity.

### 3.4. Anti-Inflammatory Effects

GLP-1 agonism also exerts anti-inflammatory effects. In HFD-fed mice, semaglutide reduced systemic inflammation by lowering TNFα, IL-6, IL-1β, and HOMA-IR [5]. In skeletal atrophy models, both semaglutide and liraglutide lowered intramuscular mRNA levels of TNFα, IL-6, and IL-1β, along with reduced levels of pro-inflammatory nuclear factor-kappa B p65 (NF-kB p65) [20,26], while semaglutide also upregulated intramuscular antioxidant defence genes (Hmox1, Nqo1, and Gstm3) [26]. Mechanistically, semaglutide increased intracellular cAMP by more than 50%, triggering two downstream pathways. It activated the protein kinase A catalytic subunit α (PKA C-α) signaling, which increased heat shock factor 1 (HSF-1) protein levels with potential cytoprotective effects. In parallel, semaglutide enhanced protein kinase B (Akt) signaling, inhibiting NF-kB phosphorylation, thereby attenuating pro-inflammatory signaling [26]. These findings suggest that GLP-1 RAs may protect skeletal muscle through combined suppression of NF-kB-mediated inflammation and enhancement of antioxidant defenses.

The anti-inflammatory and antioxidant actions of GLP-1 RAs may help protect muscle quality and function, a benefit particularly relevant in older adults, metabolically compromised patients with obesity/T2D, or those with chronic low-grade inflammation.

**Figure 1:**
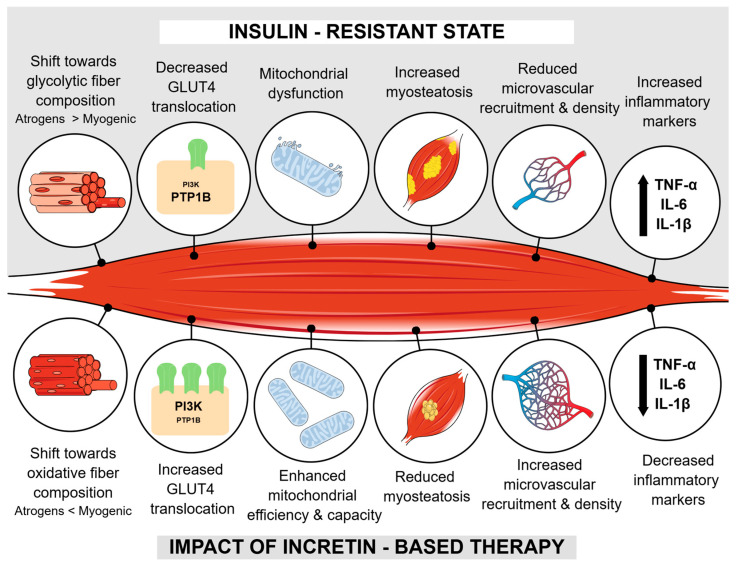
Summarized impact of incretin-based therapy on muscle health according to preclinical data. Abbreviations: GLUT4—glucose transpoter 4; PIK3—phosphatidylinositol 3-kinase; PTP1B—protein tyrosine phosphatase 1B; TNF-α—tumor necrosis factor α; IL-6—interleukine 6; IL-1β—interleukine 1β; Atrogens—MAFbx and MuRF1; Myogenic—MyoD and MyoG.

Based on available preclinical data, Figure 1 summarizes the potential impact of incretin-based therapy on various aspects of muscle health.

## 4. Impact of Incretin-Based Anti-Obesity Medications on the Skeletal Muscle—Data from Human Studies

While preclinical evidence strongly supports incretin-based therapies as modulators of muscle mass, structure and metabolism, translation to humans requires consideration of both body composition and functional outcomes. Most clinical studies have focused on body composition measurements and estimated muscle mass using non-gold standard techniques, such as BIA or DXA. These methods estimate fat-free or lean tissue across all body compartments and may overestimate true changes in skeletal muscle mass [2,38]. Recent meta-analyses of incretin-based anti-obesity medications report diverse reductions in lean body mass, measured mainly by DXA and BIA, ranging from nonsignificant decreases (less than 20%) to significant reductions of up to 30%, with comparable changes in the lean mass percentage [2,39,40,41]. Beyond the magnitude of weight reduction, lean muscle mass is also influenced by the rapidity of weight reduction, engagement in resistance training, the degree of calorie restriction and dietary protein intake [42]. Low protein consumption, particularly in older adults, or those with hypogonadism, sedentary behavior or low physical activity, may further exacerbate muscle loss, prompting recommendations of higher protein intake during active weight reduction [42].

Additionally, while these imaging modalities can indirectly monitor muscle mass, they do not capture muscle health, quality, and functional capacity, leaving important gaps in available research. The following subsections summarize the available human data for liraglutide, semaglutide, and tirzepatide on muscle health, from body composition effects to muscle preservation, functional performance, and mechanistic insights where available.

### 4.1. Liraglutide and Skeletal Muscle Health

Across multiple clinical studies, liraglutide has been used in various doses and has consistently promoted fat mass reduction with relative preservation of lean mass in individuals with obesity and/or type 2 diabetes (T2D). However, effects on muscle strength and function remain underexplored.

Several DXA-based studies demonstrate that while absolute lean mass often declines in parallel with the intensity of weight loss, the proportion of lean mass relative to body weight typically increases. For example, in a small study, Li et al. reported that in patients with T2D and obesity, 12 weeks of liraglutide 1.2 mg/day reduced both fat mass by 3.79 kg and lean mass by 1.52 kg. Treatment significantly reduced body fat percentage (from 37.9% to 35.6%) with a corresponding increase in lean mass percentage (from 62.1% to 64.4%), indicating a favorable shift in body composition [43]. Similar preservation was observed by Perna et al. in a study including 9 non-sarcopenic elderly patients with T2D (mean age 68) treated with liraglutide up to 3 mg/day over 24 weeks. While arm fat-free mass remained unchanged, leg fat-free mass increased, resulting in a median skeletal muscle index (SMI) gain of 0.03 kg/m^2^ and none of the participants developed sarcopenia [44]. In a similar randomized placebo-controlled study, liraglutide use in T2D patients also reduced visceral fat area, while subcutaneous fat area and SMI remained unchanged [45]. Similarly, in drug naïve Japanese patients, liraglutide treatment, although at a lower dose of 0.9 mg/day resulted in reduced visceral fat area and intrahepatic lipid content, improved skeletal mass index to fat mass index ratio (SMI/FMI) without a significant change in SMI [46].

Trials across diverse populations, including prediabetes [47], obesity [48], T2D on metformin [49], T1D insulin pump users [50], consistently show that liraglutide-induced weight loss is predominantly achieved by fat mass reduction, with preserved or even improved lean mass percentage.

Randomized trials also support relative lean mass preservation. In a placebo-controlled study, liraglutide 3 mg/day over 16 weeks in adults with overweight/obesity produced significant weight loss without lean mass loss [51]. In type 1 diabetes (T1D) individuals with BMI ≥ 25 kg/m^2^, six months of liraglutide treatment at a dose of 1.8 mg/day resulted in 82% of weight loss from fat mass, without significant muscle mass loss [52].

These findings were consistent even in multidisciplinary settings. In patients with overweight/obesity, liraglutide 3 mg/day combined with a multidisciplinary intervention over 1 year decreased lean body mass by 2.7 kg, representing 23% of total weight loss. This lower lean body mass loss compared to fat mass loss increased lean body percentage on follow-up [53]. In another trial, liraglutide combined with lifestyle intervention resulted in a greater weight loss than lifestyle alone, without a significant difference in fat-free mass loss after adjusting for total weight loss [54].

In more intensive interventions, liraglutide appears to support lean mass retention. A study comparing sleeve gastrectomy (SV) and intensive lifestyle modification (ILM) with or without liraglutide in patients with BMI of >40 kg/m^2^ or 35 kg/m^2^ with hypertension, sleep apnea or severe osteoarthritis, showed that liraglutide 3 mg/day together ILM resulted in 24% weight loss, almost twice greater than ILM alone and only 25% less than SV [55]. Liraglutide combined with ILM produced greater total weight loss (−26.2 kg) than ILM alone (−15.39 kg), with proportional lean body mass losses similar to ILM, but smaller than SV. Additionally, in ILM and ILM/liraglutide, most of the lean body mass loss happened during the first three months compared to the downward trend over the whole year in patients with SV [55].

Beyond body composition using BIA and DXA, some studies investigated qualitative muscle changes. In a randomized placebo-controlled trial, Pandey et al. used magnetic resonance (MRI) to evaluate thigh muscle fat, composition and volume in adults with overweight/obesity treated with liraglutide 3 mg/day over 40 weeks [37]. Liraglutide reduced tight muscle fat by approximately −0.24% over 40 weeks, countering the natural age-related increase (approximately 0.4% over 5 years), and lowered the prevalence of adverse muscle composition (a combination of low muscle mass and high fat infiltration) from 11% to 8.2%. Muscle volume fell slightly compared to placebo (−3.18% vs. −0.11%), although the change in muscle volume Z-score remained similar across both treatments, suggesting proportional reductions. The study did not, however, assess muscle strength, included predominantly women and as MRI does not distinguish between intra- and extramyocellular lipids, the impact of liraglutide on intramyocellular lipid accumulation remains unknown [37].

Metabolic and functional data are limited. In prediabetes, liraglutide 1.8 mg daily over 14 weeks and caloric restriction both improved the fat-to-lean mass ratio, with a greater reduction in the caloric restriction group, but neither intervention altered resting energy expenditure [47].

All these studies consistently show that liraglutide facilitates fat-predominant weight loss with relatively preserved lean mass, often increasing lean mass percentage and improving muscle fat infiltration. However, most data rely on imaging-based body composition measurements rather than measures of strength, performance and muscle quality, leaving the functional impact of these compositional changes unknown.

### 4.2. Semaglutide and Skeletal Muscle Health

Data from pivotal registration trials and subsequent clinical studies indicate that semaglutide produces a substantial weight loss with fat mass as the predominant contributor, though lean mass reduction is also observed. In the STEP-1 DXA substudy, semaglutide reduced total fat mass by 10.4 kg and total lean mass by 6.9 kg, corresponding to a proportional body reduction of 60% fat and 40% lean body mass (muscle and other non-fat tissues) [8].

In smaller controlled trials, the lean-to-fat loss ratio appears more favorable. A 12-week study in people with obesity receiving 1 mg/week reported a mean 5 kg body weight loss, with fat mass loss approximately three times greater than lean mass loss as assessed by air displacement plethysmography [56]. In the SUSTAIN 8 DXA substudy in patients with T2D, both semaglutide 1 mg/week and canagliflozin reduced fat and lean mass over 52 weeks, but the proportion of lean mass relative to total weight increased in both groups (−0.14% in favor of semaglutide) [57].

Bioimpedance studies also support preservation of muscle indices during semaglutide treatment. In T2D, Volpe et al. observed that semaglutide reduced fat mass index over 26 weeks, while the SMI and fat-free mass index (FFMI) declined modestly in the first 12 weeks before stabilizing. Hand grip strength test and muscle quality index (strength/muscle mass) remained unchanged [58]. Similarly, in T2D and MASLD, semaglutide treatment over 1 year lowered FMI and visceral adipose tissue with only clinically insignificant declines in FFMI and SMI. Both hand grip strength and muscle quality index remained unchanged [59].

Other short-term studies show proportional preservation of muscle mass. In Japanese patients with obesity and T2D, semaglutide reduced both fat and skeletal muscle mass over 3 months, however, skeletal muscle mass percentage remained the same. A retrospective analysis of Chinese patients receiving semaglutide 1 mg/week over 6 months resulted in a mean body weight loss of 9.9 kg (11.2%), with estimated skeletal muscle mass loss of 1.4 kg (4.8%), significantly less than estimated fat mass loss of 5.6 kg (15.6%). While total fat mass percentage significantly decreased from 41% to 37.8%, skeletal muscle mass percentage increased by 2.7% from 33.1 to 35.8% and the SMI remained unchanged. Calf circumference and grip strength were also preserved [60].

Several trials have examined semaglutide in specific populations. In HIV-positive patients with metabolic dysfunction-associated steatotic liver disease (MASLD), semaglutide 1 mg/week over 24 weeks reduced psoas muscle volume by 9.3% on MRI, nevertheless, both chair-rise time and gait speed improved, with a significant lowering of the prevalence of slow gait. This suggests maintained function despite volumetric loss [61]. In patients with T2D and MASLD, semaglutide 1 mg/week over 6 months significantly reduced skeletal muscle steatosis fraction on MRI, but did not impact skeletal muscle index (muscle cross-sectional area at L3/square of height), indicating maintenance of skeletal muscle volume [62]. In obesity with heart failure with preserved ejection fraction, semaglutide 2.4 mg weekly over 52 weeks improved both the 6-min walk test by 21.5 m and self-reported physical function [63]. In menopausal compared to premenopausal women, semaglutide 1 mg/week over 4 months produced similar proportional fat mass (4.1 ± 4.5 vs. 3.1 ± 3.7 kg; *p* = 0.3) and lean mass (0.4 ± 1.7 vs. 1.1 ± 3.7 kg; *p* = 0.1) loss in both groups, despite higher baseline adiposity in the menopausal cohort [64].

Some long-term observational data also reveal mixed effects on lean mass. In a prospective study of T2D patients with a mean age of 63, semaglutide 1 mg/week reduced fat mass by 2.1 kg over 6 months and only slightly after 12 months, while lean mass declined by over 3 kg at both 6 and 12 months. Nevertheless, both physical and mental components of the SF-36 health survey suggested improved quality of life [65].

Body composition imaging has also been used to evaluate semaglutide tissue-specific effects. Nelson et al. evaluated intrapatient computer tomography (CT)-based body composition changes after initiation of semaglutide regardless of the dose or treatment duration [66]. The group found that patients with weight loss after treatment initiation had a reduction in visceral adipose tissue and subcutaneous adipose tissue area, muscle area and liver volume, with improved liver attenuation. In contrast, patients with weight gain on the posttreatment scans showed an increase in visceral and subcutaneous adipose tissue area alongside an increase in intramuscular adipose tissue area with decreased muscle attenuation, indicative of poorer muscle quality. Half of the patients who gained weight had already stopped treatment at the time of the postsemaglutide CT scan and were therefore likely experiencing weight regain [66].

Emerging data on oral semaglutide suggests potentially better lean mass preservation, although oral semaglutide is not indicated for chronic weight loss management. In a 26-week trial of oral semaglutide 7 mg/day over 6 months in T2D, Volpe et al. confirmed a 4 kg weight loss with fat mass and FMI reduction but no fat-free mass loss. On the contrary, patients even significantly increased fat-free mass during the first three months, which was maintained thereafter [67]. In a similar retrospective study by Uchiyama et al. oral semaglutide 14 mg/day over 24 weeks in Japanese patients with T2D, reduced whole body fat and BMI without a change in lean mass or SMI. Phase angle measurement and extracellular water/total body water ratio (ECW/TBW) were also unchanged [68].

An ongoing NCT05786521 will report the impact of semaglutide 1 mg treatment on lean mass, 6-min walk distance, grip strength and short physical performance battery in older adults [69].

Overall, semaglutide promotes fat-predominant weight loss with relative preservation of lean mass and generally stable muscle strength and function when assessed. Again, most available evidence comes from body composition analysis, warranting the need for direct performance measurements.

### 4.3. Tirzepatide and Skeletal Muscle Health

Evidence from clinical trials suggests that tirzepatide produces significant weight loss while preserving a lean-to-fat loss ratio and potential improvements in muscle quality.

In the SURMOUNT-1 DXA substudy, 160 participants underwent DXA to assess the impact of tirzepatide on body composition. Pooled tirzepatide treatment resulted in 21.3% weight loss compared to 5.3% with placebo, corresponding to fat mass reductions of 33.9% versus 8.2%, and lean mass reductions of 10.9% versus 2.6%, respectively. Despite the greater body weight reduction with tirzepatide, the proportion of total body weight loss was approximately 75% for fat mass and 25% for lean mass loss, similar to placebo and consistent with many dietary restriction studies [70]. In the SURMOUNT-1 cohort, patient-reported physical function assessed (SF-36) improved compared to placebo [10].

A post-hoc exploratory analysis of the SURPASS-3 study used MRI to assess thigh muscle composition in 246 insulin-naive patients with T2D and obesity, comparing tirzepatide to insulin degludec over 52 weeks [71]. Tirzepatide significantly reduced muscle fat infiltration (−0.36 percentage points), muscle volume (−0.64 L) and muscle volume Z-score (−0.22), alongside significant weight loss. In contrast, insulin treatment caused a modest but significant increase in weight and muscle volume without a significant change in muscle fat infiltration or muscle volume Z-score. Compared with UK Biobank population-based estimates, muscle volume reduction with tirzepatide treatment closely followed the expected changes for the degree of weight loss. At the same time, the decrease in muscle fat infiltration was 2–4 times greater than the typical annual increase seen with aging, indicating that tirzepatide is likely not associated with accelerated muscle loss and may have a targeted, positive effect on muscle quality [71].

Interventional studies combining tirzepatide with exercise suggest potential additive benefits. In obese men, 6 weeks of combined resistance and aerobic training with either placebo, tirzepatide 2.5 mg or tirzepatide 5 mg compared to placebo and tirzepatide alone, significantly decreased BMI, fat mass, lipids, HOMA-IR and fasting blood glucose and significantly increased muscle strength (chest and leg press) [72].

In more vulnerable populations, tirzepatide also appeared to be muscle-sparing. In 9 patients with diabetic kidney disease on hemodialysis, tirzepatide doses of 2.5–7.5 mg reduced dry weight and BMI without decreasing skeletal muscle mass, SMI or extracellular/body weight ratio ECW/BW (a marker of protein-energy wasting) [73].

Other studies indicate potential lean mass preservation or gain in specific settings. In males with metabolic hypogonadism, tirzepatide treatment over two months improved hormonal status, reduced body weight, waist circumference, BMI and fat mass more than testosterone and non-pharmacologically treated. In terms of lean mass change over two months, the tirzepatide group experienced an average increase of +17.9% (−1.3%; +41.9%), the testosterone group an average increase of +10.5% (+2.2%; +15.6%), in contrast to the lifestyle-only or placebo group, which showed no gain in lean mass (0.0%) [74]. In another study, patients with obesity received tirzepatide 5 mg for 12 weeks alongside either a low-calorie diet (LCD) defined as 1200 kcal/day with 50% carbohydrates, 20% protein and 30% fat or low-energy ketogenic therapy (LEKT), defined as 1200 kcal/day with less than 30 g of carbohydrates, 44% fat and 43% protein or 1.3 g/kg ideal body weight. Although both groups had a similar significant reduction in body weight around 10%, the group receiving LEKT showed greater fat mass loss (−13.4% vs. −10.2%) with a lesser decrease in fat-free mass (−0.50% vs. −4.29%) and a lesser reduction in muscle strength (−0.3% vs. −4.1%). The resting metabolic rate was significantly decreased only in the group receiving LCD (−5.3%). This study implied that ketogenic therapy may have a role in maintaining muscle integrity and preventing metabolic adaptation [75]. This aligns with recent nutritional guidance, recommending higher protein intake during active weight loss to preserve lean mass. Suggested targets include 1.2–1.6 g/kg per day or 1.5 g/kg of lean body mass when body composition data are available. Alternatively, for practical adherence, an absolute protein target of 80–120 g per day or 16–24% of total energy on a 2000 kcal/day diet is also reasonable for most adults [42].

A forthcoming trial will evaluate whether personalized weekly lifestyle coaching with an emphasis on protein intake and resistance training for 6 months, compared to standardized health counseling, will enhance body composition and strength preservation compared to standardized health counseling in obese patients treated with tirzepatide, using DXA and composite strength testing [76].

Overall, current human data indicate that tirzepatide-induced weight loss follows a physiological lean-to-fat loss ratio and, in several contexts, may improve muscle quality by reducing intramuscular fat. Ongoing trials incorporating advanced imaging and functional testing will be essential to confirm whether tirzepatide can actively support muscle health.

## 5. Limitations, Considerations and Future Directions

Interpretations of current findings are limited by several confounding factors that may strongly influence skeletal muscle mass and quality, making it difficult to attribute observed effects exclusively to incretin-based therapies.

Dietary protein intake, habitual physical activity, and resistance training are well-established modulators of lean body mass and muscle function. Low protein intake or sedentary behavior exacerbates lean mass loss during weight reduction, while higher protein intake and structured resistance training can help preserve muscle integrity [42].

Additionally, concurrent medications may also impact muscle outcomes. For instance, metformin, often used in diabetes, has been reported to modulate mitochondrial function [77,78] and, in some studies, to blunt resistance training-induced muscle hypertrophy in older adults [79]. Sodium-glucose cotransporter-2 (SGLT-2) inhibitors, often used in diabetes, chronic renal disease or heart failure, reduce body weight and fat mass but are also associated with modest decreases in lean mass [80,81]. However, in patients with heart failure with reduced ejection function, SGLT-2 inhibition was also linked with decreased myofiber atrophy and larger oxidative type I fibers, alongside anti-inflammatory and pro-metabolic skeletal muscle adaptations [82]. Supporting evidence from preclinical models also shows that SGLT-2 inhibition may preserve muscle function in heart failure rats [83], ameliorate age-dependent muscle atrophy in diabetic mice [84], and reduce age-related muscle fibrosis in aging mice [85].

The specific contribution of incretin-based therapies to muscle health must therefore be interpreted within the broader context of lifestyle factors, background pharmacotherapy, and comorbidities, emphasizing the need for well-controlled mechanistic studies that integrate standardized nutrition, structured exercise protocols, and the influence of concurrent therapies.

Future studies should bridge the translational gap through mechanistically rich, functionality-oriented, and adequately powered clinical trials to fully evaluate the role of incretin-based therapies in muscle health. Studies should prioritize mechanistic human investigation by incorporating muscle biopsies, transcriptomic and proteomic profiling to confirm molecular pathways identified in preclinical models. Alongside imaging-based muscle mass measurement, future studies should also assess functional outcomes by measuring muscle strength, endurance and performance. The long-term effect of incretin-based therapy on muscle health should be evaluated, especially in older adults, populations with increased sarcopenic risk and chronic disease. Studies should also investigate incorporating structured resistance training and optimized protein intake to maximize muscle preservation as well as evaluate whether emerging pharmacological candidates, such as selective androgen receptor modulators (SARMs) and antimyostatin agents, with targeted anabolic effects on skeletal muscle, may complement the metabolic effects of incretin therapies.

## 6. Conclusions

Preclinical evidence consistently demonstrates that incretin-based anti-obesity medications such as GLP-1 RAs may have direct positive effects on skeletal muscle beyond their roles in glucose regulation and weight loss. These agents attenuate muscle atrophy, enhance myogenesis, improve myofibrillar architecture, reduce myosteatosis, increase mitochondrial capacity, mitigate oxidative stress and inflammation, increase muscle capillary recruitment and modulate key molecular pathways involved in muscle metabolism and regeneration. While most human data have focused on body composition analysis and indirect measurements of muscle mass loss, recent imaging studies using MRI and CT provide early translational evidence of improved muscle quality, demonstrated by reduced intramuscular fat and preservation of muscle strength in several cohorts. Future studies should prioritize using gold-standard imaging and functional assessments, integrate standardized resistance training and protein intake protocols, and investigate molecular pathways to clarify whether the preclinical benefits translate into meaningful preservation of muscle quality and performance across diverse populations, particularly those with high risk of sarcopenia.
